# Gingival changes after removal of fixed orthodontic appliances: a prospective cohort clinical study

**DOI:** 10.1590/2177-6709.30.3.e252531.oar

**Published:** 2025-10-20

**Authors:** Luciana Cláudia Diniz TAVARES, Luis Carlos GOUVÊA, Alfredo CHAOUBAH, Gustavo Silva MAXIMIANO, Jocimara Domiciano Fartes de Almeida CAMPOS, Sergio Luiz MOTA, Robert Willer Farinazzo VITRAL, Marcio José da Silva CAMPOS

**Affiliations:** 1 Federal University of Juiz de Fora, School of Dentistry, Department of Orthodontics (Juiz de Fora/MG, Brazil).; 2 Federal University of Juiz de Fora, School of Exact Sciences, Department of Statistics (Juiz de Fora/MG, Brazil).; 3 Estácio de Sá Juiz de Fora University Center, School of Dentistry, Department of Periodontics (Juiz de Fora/MG, Brazil).

**Keywords:** Orthodontics, Periodontics, Interdental papilla, Gingival hyperplasia, Ortodontia, Periodontia, Papila interdental, Hiperplasia gengival

## Abstract

**Introduction:**

The presence of orthodontic brackets can compromise the efficiency of oral hygiene, leading to changes in oral microbiota, increasing the risk of gingival changes after removal of the fixed orthodontic appliance.

**Objective:**

The objective of this study was to evaluate changes in gingival volume in the lower anterior teeth after removal of the fixed orthodontic appliance.

**Methods:**

The sample consisted of 84 lower anterior teeth of 14 adult individuals in the final phase of fixed orthodontic treatment and had the gingival condition of their lower incisors recorded through photographs. The evaluation times were from T0 to T4. At T0, the individuals underwent removal of dental calculus and prophylaxis with a sodium bicarbonate jet, installation of a fixed retainer, digital periapical radiography of the anteroinferior teeth and extraoral frontal photograph to record the gingival condition. At T1, the appliance was removed and the gingival biotype was determined. Other photographic exams were performed at times T2, T3 and T4.

**Results:**

Clinical incisor crown values showed a significant increase 21 days after orthodontic appliance removal. The height of the papilla between the incisors showed a significant reduction on the 14th day after removal of the appliance, while the papilla between the canine and the lateral incisor showed significance only in the last evaluation.

**Conclusions:**

The sample showed a predominance of thin gingival biotype, the radiographic variables showed no significant correlation. There were gingival changes in the anteroinferior region, with spontaneous and progressive reduction of all analyzed papillae.

## RESUMO

**Introdução:**

A presença de braquetes ortodônticos pode comprometer a eficiência da higiene bucal, levando a alterações na microbiota bucal, aumentando o risco de alterações gengivais após a remoção do aparelho ortodôntico fixo.

**Objetivo:**

O objetivo deste estudo foi avaliar as alterações no volume gengival nos dentes anteroinferiores após a remoção do aparelho ortodôntico fixo.

**Métodos:**

A amostra foi composta por 84 dentes anteroinferiores de 14 indivíduos adultos em fase final de tratamento ortodôntico fixo e que tiveram a condição gengival de seus incisivos inferiores registrada por meio de fotografias. Os tempos de avaliação foram de T0 a T4. Em T0, os indivíduos foram submetidos à remoção do cálculo dentário e profilaxia com jato de bicarbonato de sódio, instalação de contenção fixa, radiografia periapical digital dos dentes anteroinferiores e fotografia frontal extrabucal para registro da condição gengival. Em T1, o aparelho foi removido e o biotipo gengival foi determinado. Outros exames fotográficos foram realizados nos tempos T2, T3 e T4.

**Resultados:**

Os valores clínicos da coroa dos incisivos apresentaram aumento significativo 21 dias após a remoção do aparelho ortodôntico. A altura da papila entre os incisivos apresentou redução significativa no 14º dia após a remoção do aparelho, enquanto a papila entre o canino e o incisivo lateral apresentou significância apenas na última avaliação.

**Conclusões:**

A amostra apresentou predomínio de biotipo gengival fino e as variáveis radiográficas não apresentaram correlação significativa. Houve alterações gengivais na região anteroinferior, com redução espontânea e progressiva de todas as papilas analisadas.

## INTRODUCTION

The establishment of adequate occlusal relationships through orthodontic treatment allows a favorable oral health condition,^1^ increasing the longevity and quality of the dentition^2^ and contributing to a harmonious facial aesthetics.^3^

A correctly indicated and conducted orthodontic treatment associated with efficient oral hygiene is considered a treatment without risk to periodontal tissues.^4^ However, the presence of brackets and orthodontic bands can compromise the efficiency of oral hygiene,^5^ leading to significant changes in bacterial plaque^6^ ^,^ ^7^ and of the oral microbiota, increasing the risk of gingival changes.^5^ ^,^ ^8^

The presence of the multibracket appliance has been related to some soft tissue problems, such as cheek mucosal lesions, gingival recessions and gingivitis.^9^ Among gingival alterations, gingival hyperplasia is one of the most frequent. This condition is characterized by the growth of the gingival tissue, mainly of the interdental papillae which acquire a flaccid consistency and erythematous color, being induced by the accumulation of bacterial biofilm^10^ or by the release of ions resulting from the corrosion of orthodontic appliances.^11^

Generally, the gingival tissue returns to its normal volume after the removal of the orthodontic appliance and maintenance of correct oral hygiene,^12^ resulting in a continuous reduction of the probing depth up to 6 months after the removal.^13^ ^,^ ^14^ However, the hyperplastic condition of the gingival tissue may persist after the end of treatment due to the presence of periodontopathogens, compromising the aesthetics and making oral hygiene difficult, requiring periodontal surgical procedures such as gingivoplasty or gingivectomy.^10^ ^,^ ^15^ However, there is evidence that the volume of gingival tissue returns to normal after removal of the orthodontic appliance and maintenance of correct oral hygiene,^12^ as demonstrated in our study, within a period of 21 days.

The aim of this study was to evaluate changes in gingival volume that spontaneously occurred in the anteroinferior teeth after removal of the fixed orthodontic appliance. The hypothesis is that the gingival volume spontaneously decreases after the removal of the orthodontic appliance during the evaluation period.

## MATERIAL AND METHODS

This prospective observational cohort study was approved by the Committee of Ethics and Research in Human Beings of the Federal University of Juiz de Fora (number 3.292.094) and all subjects participated voluntarily. The sample size calculation for a power test of 80% and a significance level of 5% (bilateral), assuming a mean of differences of 0.11mm with a standard deviation of 0.32mm, indicated a sample equal to or greater than 69 teeth. The sample size calculation was aimed at teeth and not subjects, as we understand that local factors such as the configuration of the alveolar bone, the morphology of adjacent proximal surfaces and also the volume and shape of tooth roots may be as or more important than individual factors in establishing the researched effect.

The final sample consisted of 84 anteroinferior teeth (56 incisors and 28 canines) from 14 adults with an average age of 32.7 years old, being 5 men (24.4 years old) and 9 women (40.66 years old). Participants were recruited in a private clinic and in the Orthodontic clinics of the Federal University of Juiz de Fora, between May 2019 and February 2020 and were all in the process of completing their orthodontic treatment with edgewise fixed appliances in both dental arches and there was no clinical and/or radiographic evidence of periodontal disease (absence of bleeding on probing, gingival redness or loss of attachment) in any of the lower anterior teeth.

In the first stage of the study (T0), the subjects underwent removal of dental calculi and prophylaxis with a jet of sodium bicarbonate, aiming at completely cleaning the dental crowns, and a 0.018-in TwistFlex wire retainer was fixed to the lower canines and incisors. The retainer was maintained with the orthodontic appliance until T1 to prevent unwanted tooth movement in the event of retainer debonding. Still at T0, digital periapical radiography of the lower anterior teeth and frontal extraoral photography were performed to record the gingival condition.

Digital periapical radiography (Scanner VistaScan, Durr Dental BR) was performed with Rinn XCP radiographic positioner (Dentsply, USA) with the center of the sensor positioned between the right and left lower central incisors and the central axis of the X-ray beam directed to the same point with exposure time of 0.25s.

The photography was carried out with a resolution of 4000x3000 pixels with the aid of an expandex lip retractor (Double model, Indusbello, Londrina, Brazil) and under artificial light. Each individual was sitting in an upright position and the camera (Smartphone Samsung Galaxy, 12-megapixel camera) set on the midline, 15 cm from the mandibular central incisors, at the height of the occlusal plane. All camera parameters and lighting conditions were kept constant to obtain all photographic images.

In order to standardize the oral hygiene process, all participants received an oral hygiene kit, containing dental floss, 1,500 ppm fluoride toothpaste and a toothbrush (Colgate-Palmolive^®^, São Paulo, Brazil) and were instructed to use only material received with the Bass technique^16^ during the evaluation period.

Approximately three weeks after T0 (20.8 ±1.897 days), the lower orthodontic appliance was removed (T1) and a new photograph was taken. At this time, the gingival biotype was determined through clinical examination,^17^ ^,^ ^18^ in which the transparency of the tip of the periodontal probe inserted into the gingival sulcus of the right lower central incisor was evaluated and the biotype classified as thin (visible probe tip) or thick (non-visible probe tip).^19^ Other photographic examinations were performed at T2 (27.7 ± 2.050 days), T3 (34.6 ± 2.195 days) and T4 (42.0 ± 1.852 days), resulting in a total of five photographic records of each individual.

All photographic and radiographic images were analyzed with the ImageJ 1.53c software (National Institutes of Health, USA). To correct possible dimensional distortions related to the photographic taking, the measurements obtained in the photographs were corrected using the mesiodistal dimension of the incisal edge of the right mandibular central incisor obtained clinically (in millimeters) with the aid of a digital caliper (Starrett, Itu, Brazil), and digitally in the ImageJ software (in pixels) through the rule of three.

In the photographic images, the clinical crown (CC) and the interdental papillae (IP) were measured. In the radiographic images, the alveolar bone crest (BC), the width of the interproximal space (IS-w), the height of the interproximal space (IS-h) and the area of the interproximal space (IS-a) were determined. All measurements were evaluated by the same examiner (L.C.D.T.).

**Figure 1:**
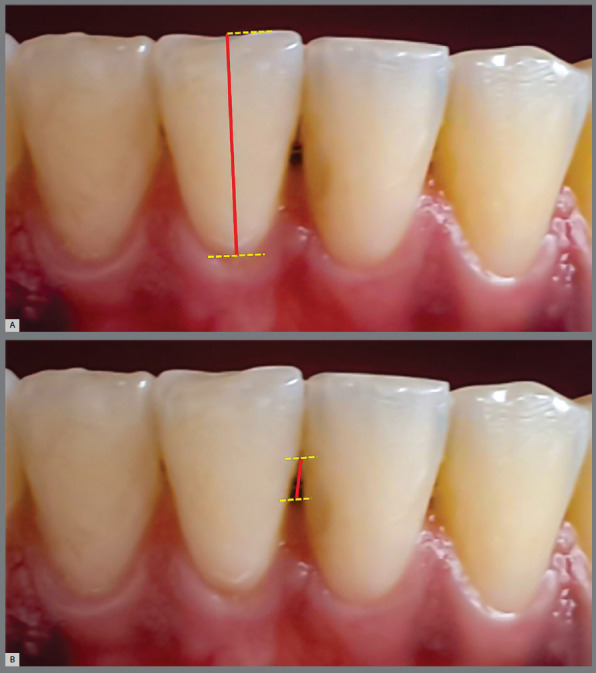
Intraoral photography determining the clinical crown (**A**, red line) and the Interdental Papilla (**B**, red line).

Clinical crown (CC): the height of the clinical crown of canines and incisors was determined by the distance between the orthogonal projections of the most incisal point of the incisal edge and of the most cervical point of the dental crown on the long axis of the dental crown (Fig. 1A).

Interdental papilla (IP): defined by the distance between the most incisal point of the contact surface between two adjacent teeth and the most incisal point of the interdental papilla (Fig. 1B). The increase in this distance represents the reduction in the height of the papilla. The papillae located between the lower incisors (3 papillae) and between the canines and lateral incisors (2 papillae) were evaluated.

The measurements evaluated in radiographic images (BC, IS-w, IS-h and IS-a) were determined in three interproximal spaces, located between right lateral and central incisors; right and left central incisors; left central and lateral incisors.

**Figure 2:**
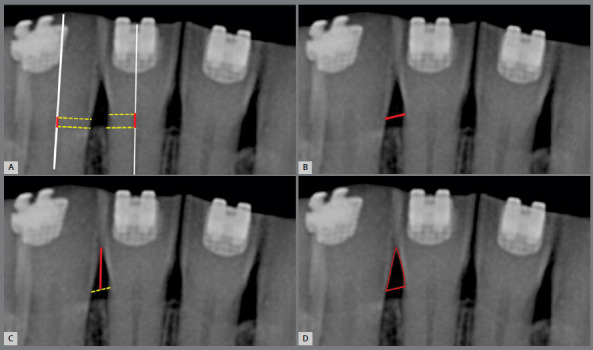
Determination of interproximal radiographic measurements of: height of interproximal bone (A), width of interproximal space (B), height of interproximal space (C) and area of interproximal space (D), represented by red lines.

Alveolar bone crest (BC): determined by the distance between the orthogonal projections of the most incisal point of the BC and of the cementoenamel junction on the long axis of the crown of the evaluated incisor^20^ (Fig. 2A). Three interproximal regions were defined: 1) right - arithmetic mean of the bone crests heights evaluated on the mesial surface of the right lateral incisor and distal surface of the right central incisor; 2) central - arithmetic mean of the bone crests heights evaluated on the mesial surfaces of the right central incisor and of the left central incisor; 3) left - arithmetic mean of the bone crests heights evaluated on the distal surface of the left central incisor and mesial surface of the left lateral incisor.

Interproximal space width (IS-w): distance between the cementoenamel junctions of the proximal faces of two adjacent teeth (Fig 2B).

Height of the interproximal space (IS-h): distance between the most cervical point of the contact surface and the center of line joining the cementoenamel junctions of the proximal faces of adjacent teeth (Fig 2C).

Area of the interproximal space (IS-a): area between a line joining the cementoenamel junctions of the adjacent proximal surfaces, the most cervical point of the contact surface of the teeth and the proximal contours of adjacent teeth (Fig 2D).

In order to calibrate the examiner and calculate the method error, the gingival biotype determination and measurements of all photographic and radiographic variables, in T0 of 4 subjects, were performed twice within a 20-day interval.

### STATISTICAL ANALYSIS

Intra-examiner reproducibility was assessed with the Intraclass Correlation Coefficient (ICC). The distribution pattern of the clinical crown and interdental papilla variables were determined using the Kolmogorov-Smirnov test. The mean values of clinical crown and interdental papilla were compared in between the evaluation times (T1-T0, T2-T1, T3-T2, T3-T1, T4-T3 and T4-T1) using Student’s t-test for paired samples and between gingival biotypes with Student’s t-test for independent samples. The correlations between the changes (T4-T1) of the clinical crown and the interdental papilla of the incisors and the radiographic variables were evaluated using Pearson’s correlation. For all tests, a confidence interval of 95% and significance of 5% were considered, with all data processed with the SPSS Statistics 20.0.0 software (SPSS, Chicago, USA).

## RESULTS

The intra-examiner reliability was considered excellent,^21^ with an ICC ≥ 0.844. All variables presented a normal distribution pattern in the evaluated sample (p-value < 0.05).

**Figure 3:**
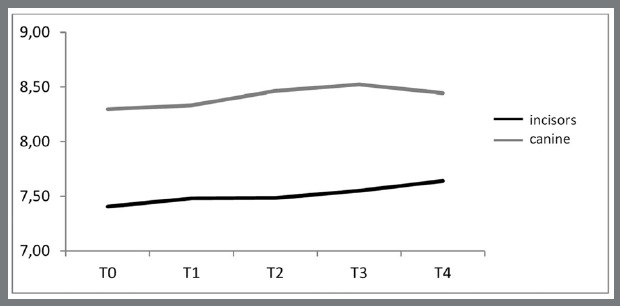
Behavior of incisor and canine clinical crowns over the evaluation times.

**Figure 4:**
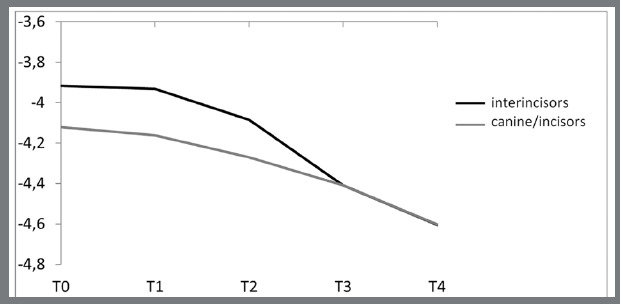
Behavior of the interdental papillae (interincisors and canine/incisors) over the evaluation times (mean values were multiplied by -1 to facilitate graphic visualization).

Table 1 and Figure 3 and 4 show the mean values of clinical crown and papilla in incisors and canines over the 5 evaluation times. Overall, clinical crowns increased, and interdental papillae progressively decreased during the evaluation period.

**Table 1:** Mean values of clinical crowns and interdental papillae at evaluation times.

	T0	T1	T2	T3	T4
	Mean (SD)	Mean (SD)	Mean (SD)	Mean (SD)	Mean (SD)
Time (days)	0	20.79 (1.897)	27.71 (2.050)	34.57 (2.195)	42.00 (1.852)
Clinical crown (mm)					
Incisors	7.41 (1.259)	7.48 (1.202)	7.49 (1.194)	7.55 (1.071)	7.64 (1.173)
Canines	8.30 (1.801)	8.33 (1.784)	8.46 (1.609)	8.52 (1.618)	8.44 (1.750)
Interdental papilla (mm)					
Interincisors	3.92 (1.084)	3.93 (0.961)	4.08 (0.757)	4.43 (0.962)	4.60 (1.113)
Canine / incisor	4.12 (1.141)	4.16 (1.285)	4.27 (1.001)	4.34 (1.361)	4.60 (1.561)

SD = standard deviation.

The values of clinical crown of incisors only showed a significant increase (0.16mm/2.13%) between periods T4 and T1, twenty-one days after removal of the orthodontic appliance. In the canines, however, no significant alteration was identified (Table 2).

**Table 2:** Mean values of changes in clinical crowns and heights of interdental papillae between the evaluation periods.

	T1-T0	T2-T1	T3-T2	T4-T3	T3-T1	T4-T1
Clinical crown (mm)						
Incisors	0.07	0.01	0.06	0.09	0.07	0.16*
Canines	0.03	0.13	0.06	-0.08	0.19	0.11
Interdental papilla (mm)						
Interincisors	0.01	0.15	0.35*	0.17*	0.51*	0.67*
Canine / incisor	0.04	0.11	0.07	0.26	0.18	0.44*

* significant difference (p<0.05) between two periods, according to the Student’s t-test for paired samples.

The height of the papilla between incisors showed a significant reduction on 14th day after the removal of the orthodontic appliance (T3), resulting in a total reduction of 17.11% in the period evaluated. On the other hand, the papilla between canine and lateral incisor only showed a significant reduction by the last assessment (T4), with a total change of 10.60% (Table 2).

**Figure 5:**
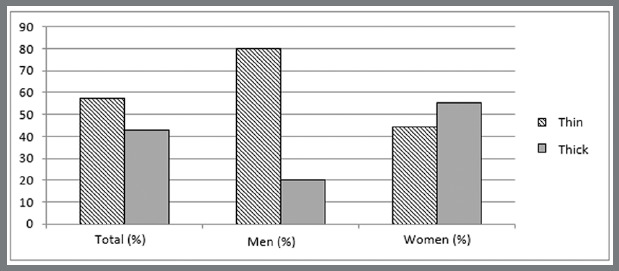
Distribution of gingival biotypes.

The sample evaluated showed a small predominance of thin gingival biotype (57.14%). Among men, 80.0% had a thin biotype, while 55.5% of women had a thick biotype (Fig 5).

The behavior of the clinical crowns and interdental papillae, as well as the subjects’ age, were compared within the gingival biotypes (Table 3). Although the incisors were the only ones showing a significant difference, subjects with thick biotype showed greater changes in the evaluation period.

**Table 3:** Mean values of changes (T4-T1) in crown and papillary height, grouping teeth according to gingival biotype

	Thick biotype		Thin biotype		p-value*
	n	Mean (SD)	n	Mean (SD)	
Age (years)	6	36.83 (20.034)	8	29.6 (12.727)	0.425
Clinical crown (mm)					
Incisors	24	0.28 (0.369)	32	0.06 (0.388)	0.041
Canines	12	0.24 (0.507)	16	0.01 (0.352)	0.159
Interdental papilla (mm)					
Interincisors	18	0.87 (0.871)	24	0.38 (0.702)	0.025
Canine / incisor	12	0.86 (0.788)	16	0.24 (0.709)	0.149

* Student’s t-test for independent samples; p-value ≤ 0.05 means significant difference.

The radiographic variables evaluated in the interproximal spaces (BC, IS-w, IS-h and IS-a) did not present a significant correlation with the observed clinical changes between T1 and T4 (Table 4).

**Table 4:** Correlation between the radiographic variables of the interproximal space and the changes of the clinical crown and the height of the papilla of the incisors (T4-T1).

	Average (SD)	Clinical crown T4-T1		Interincisor papilla T4-T1	
		Pearson correlation	p-value	Pearson correlation	p-value
Bone crest - BC (mm)	1.78 (1.149)	0.019	0.903	-0.205	0.193
Interproximal space					
Width - IS-w (mm)	1.62 (0.474)	0.263	0.092	0.151	0.340
Height - IS-h (mm)	3.38 (0.797)	0.008	0.961	0.053	0.738
Area IS-a (mm^2^)	2.02 (1.330)	0.140	0.377	0.105	0.508

SD= standard deviation.

## DISCUSSION

Orthodontic treatment aims to correct malocclusions, eliminating occlusal imbalances and facilitating oral hygiene.^22^ However, the installation of fixed orthodontic appliances is accompanied by greater difficulty in cleaning the teeth, which can lead to inflammation of the gingival tissue,^23^ ^-^ ^25^ characterized by the presence of bleeding on probing and increasing the volume of the gingiva, especially in the lower anterior teeth,^26^ caused by the significant increase in pathogens that cause gingival inflammation.^23^ ^,^ ^26^

Despite being a dental plaque retention agent, the fixed retainer installed on the lower anterior teeth after removing orthodontic appliances is associated with maintenance^27^ or progressive improvement^13^ ^,^ ^14^ of local periodontal condition in the short term. After installation of fixed retainers, the behavior of periodontal tissues may vary according to the material used,^14^ and it was necessary to standardize it in the present study.

As demonstrated in this prospective cohort study, despite the gingival tissues showing signs of inflammation and swelling due to the presence of the orthodontic appliance,^23^ ^-^ ^26^ there is evidence that after removal of the fixed appliance, and consequent interruption of the offending agent, the tissues recover spontaneously and gradually return to the normal condition,^12^ ^,^ ^25^ ^,^ ^28^ ^,^ ^29^ provided that the individual maintains an adequate standard of oral hygiene.^12^ ^,^ ^25^ Therefore, it seems reasonable that periodontal surgeries such as gingivoplasty and gingivectomy should be postponed until the tissues have stabilized their volume after completion of orthodontic treatment.

The findings in the present study indicated a distinct behavior between the gingival tissues associated with incisors and canines. Significant changes in the size of the clinical crown were observed along the evaluation period only in the mandibular incisors, and the papillae located between the incisors showed a significant reduction 7 days before the papillae located between the lateral incisors and canines. This difference may have occurred due to the fact that the papilla follows the shape of the adjacent dental crowns and as the canine has a more robust crown, its papillae are taller and more voluminous, which may have influenced the time when volume changes were identified.^30^

In the evaluated sample, 5 of the 6 subjects with thick gingival biotype were women and, in general, this gender had a thick biotype in 55% of cases, contrary to the tendency of women to present thin gingival biotype.^31^ In addition, despite the fact that young people present a prediction of thick biotype in the anteroinferior region,^19^ in the evaluated sample, no significant difference was identified concerning the age of subjects with thin and thick biotypes. The method of evaluating the gingival biotype through periodontal probe transparency is considered simple, fast and efficient.^19^ However, the divergence of results demonstrates that the assessment of the biotype still needs controlled studies in order to determine a more accurate classification pattern.

In this study, gingival tissues of thick biotype showed greater changes than tissues of thin biotype, being significant only in the lower incisors. This difference can be explained by the fact that the thick periodontium presents a more intense hyperplastic response to the presence of bacterial plaque,^17^ in addition, the thick biotype is related to short teeth and, consequently, smaller interdental papillae that respond more quickly to the removal of the etiologic agent of hyperplasia.^31^

It has been demonstrated that factors associated with dental anatomy such as crown shape, interradicular distance^30^ and the distance between the bone crest and the point of contact determine the shape and volume of the gingiva and can influence gingival behavior against inflammatory stimuli, regardless of gender and age of the subjects.^30^ In the present study, although gingival alterations were not significantly related to the dimensions of the interproximal space, it was observed a tendency for the clinical crown to undergo greater modifications in wider interproximal spaces.

Clinically, in the face of permanent hyperplastic changes in gingival volume after the end of orthodontic treatment, surgical procedures of gingivectomy or gingivoplasty should be indicated aiming to reduce gingival inflammation, facilitating teeth hygiene^22^ and favoring esthetics.^1^ However, in the present study, the periodic evaluations carried out after the removal of the orthodontic appliance indicated a progressive reduction in gingival volume without the implementation of any specific therapeutic approach possibly due to the reduction of bacterial plaque located on the dental surfaces.^5^ ^,^ ^15^ ^,^ ^24^ ^,^ ^26^ Thus, it seems reasonable that invasive procedures to reduce gingival volume should be postponed for at least twenty-one days after the removal of the orthodontic appliance.

The final evaluation time of the subjects was a limitation of the study since the gingival tissues were expected to be observed until they presented volume stabilization. However, due to the interruption of clinical activities imposed by health regulations to contain the COVID-19 pandemic, the monitoring of subjects had to be interrupted and the final observation time was 21 days after removal of the orthodontic appliance. Further studies must be carried out in order to determine the period between the removal of the orthodontic appliance and the end of relevant volume reduction of the gingival tissue, indicating the ideal time to perform surgical procedures.

## CONCLUSION

After removal of the fixed appliance, gingival changes occurred in the anteroinferior region, with a spontaneous and progressive reduction in all analyzed interdental papillae and increase in clinical crown. However, such alterations were not related to the biotype and radiographic characteristics of the interproximal spaces.
